# The Antimicrobial Effect of Cold Atmospheric Plasma against Dental Pathogens—A Systematic Review of In-Vitro Studies

**DOI:** 10.3390/antibiotics10020211

**Published:** 2021-02-20

**Authors:** Gert Jungbauer, Dominick Moser, Steffen Müller, Wolfgang Pfister, Anton Sculean, Sigrun Eick

**Affiliations:** 1 Department of Periodontology, School of Dental Medicine, University of Bern, 3010 Bern, Switzerland; dominick.moser@bluewin.ch (D.M.); anton.sculean@zmk.unibe.ch (A.S.); sigrun.eick@zmk.unibe.ch (S.E.); 2 Department of Cranio-Maxillofacial Surgery, Hospital of the University of Regensburg, 93053 Regensburg, Germany; steffen.mueller@ukr.de; 3 Department of Hospital Hygiene, Sophien- und Hufeland-Klinikum Weimar, 99425 Weimar, Germany; W.Pfister@klinikum-weimar.de

**Keywords:** cold atmospheric plasma, non-thermal plasma, antimicrobial, dentistry, *Streptococcus mutans*, *Enterococcus faecalis*, *Candida albicans*, periodontal disease, peri-implant disease, in vitro

## Abstract

Interest in the application of cold atmospheric plasma (CAP) in the medical field has been increasing. Indications in dentistry are surface modifications and antimicrobial interventions. The antimicrobial effect of CAP is mainly attributed to the generation of reactive oxygen and reactive nitrogen species. The aim of this article is to systematically review the available evidence from in-vitro studies on the antimicrobial effect of CAP on dental pathogens. A database search was performed (PubMed, Embase, Scopus). Data concerning the device parameters, experimental set-ups and microbial cultivation were extracted. The quality of the studies was evaluated using a newly designed assessment tool. 55 studies were included (quality score 31–92%). The reduction factors varied strongly among the publications although clusters could be identified between groups of set pathogen, working gases, and treatment time intervals. A time-dependent increase of the antimicrobial effect was observed throughout the studies. CAP may be a promising alternative for antimicrobial treatment in a clinically feasible application time. The introduced standardized protocol is able to compare the outcome and quality of in-vitro studies. Further studies, including multi-species biofilm models, are needed to specify the application parameters of CAP before CAP should be tested in randomized clinical trials.

## 1. Introduction

The application of physical plasma in medicine is a promising tool for disinfection and therefore a lot of data has been published within the last decade. Developed to non-chemically decontaminate in biomedical and food industries, cold atmospheric plasma (CAP) has emerged in the medical field. Plasma, often referred to as the ‘fourth state of matter’, is an ionized gas with approximately neutral charge. Terms such as non-thermal and atmospheric pressure plasma further describe the nature of the physical plasma, which operates under atmospheric pressure and does not exceed 50 °C and is therefore tolerable for human tissues. Plasma sources can be divided into indirect and direct plasma devices. In an indirect plasma device, commonly constructed as a jet design, plasma is generated by ionization of the working gas between two electrodes and constantly pushed out by the gas flow. In a direct plasma device—such as a plasma brush, volume dielectric barrier discharge (VDBD), or corona discharge device—plasma is generated between the electrode of the device and the treated surface, which functions as a grounded electrode. These devices can operate by utilizing ambient air [1].

Indications of CAP application in dentistry are: bonding to dentin and ceramics, curing of composites, bleaching, surface activation of dental implants, and antimicrobial therapeutic interventions in cariology, endodontology, periodontology, and implantology [2]. The microbicidal properties are based on the generation of reactive oxygen (ROS) and nitrogen species (RNS) including free radicals, charged particles, electric fields, and electric radiation such as UV radiation. Subsequently, these physical processes lead to antimicrobial effects, most probably caused by oxidation of cell membranes and DNA [3]. Various structures of the microbes are targeted by ROS and RNS, the cell wall is etched, and the membrane is damaged by disruption and lipid peroxidation. The bacterial DNA and RNA are impaired by oxidative damage, base modification, and strand breaks. Furthermore macromolecules, like proteins may become unfolded or modified [3]. Most dental treatments are aiming to remove or disrupt the oral biofilms. An ecological shift towards an anaerobic environment, especially when an adequate removal of the biofilm is hampered, leads to an establishment of anaerobic bacteria within the biofilm [4]. In the initial biofilm formation on the tooth surface, non-mutans streptococci—like *Streptococcus mitis* and *Streptococcus sanguinis*—are early colonizers causing acidification and introduction of more cariogenic microorganisms like *Streptococcus mutans* [5]. Lactobacilli, especially the subspecies *L. acidophilus, L. rhamnosus, L. casei*, and *L. fermentum* are often found in active carious lesions and therefore highly associated with increased caries activity [6]. *Enterococcus faecalis* is commonly known in the context of hospital acquired infections. In dentistry, this Gram-positive bacterium is frequently identified in reinfected root canals [7]. *Porphyromonas gingivalis* and *Aggregatibacter actinomycetemcomitans* are Gram-negative, anaerobic rods highly associated with periodontal disease [8]. *P. gingivalis* is capable in breaking down the host immunotolerance and is therefore referred to as the keystone-pathogen in periodontitis [9]. Some yeast strains, mainly *Candida albicans*, are opportunistic pathogens found in oral infections. Primarily known for causing denture stomatitis, *C. albicans* can also be isolated from infected root canals and periodontal or peri-implant pockets, especially in immune compromised patients.

To the best of our knowledge, until now no systematic review has attempted to analyze the available literature on the potential antimicrobial effects of the in-vitro application of cold atmospheric plasma. Therefore, the aim of this systematic review was to analyze the available literature regarding the antimicrobial effects of in-vitro application of cold atmospheric plasma on various types of oral microorganisms.

## 2. Methods

### 2.1. Search Strategy

**Figure 1:**
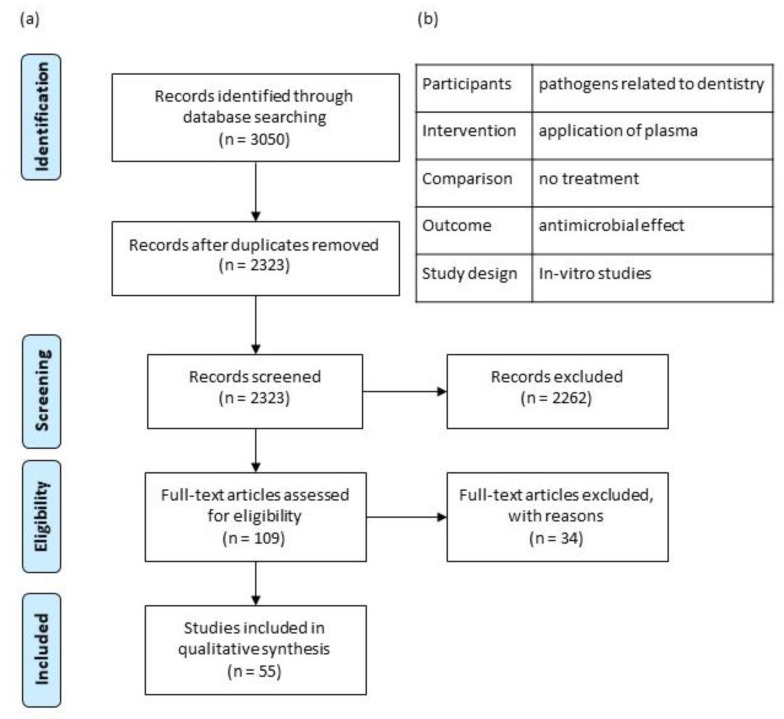
(**a**) Flow diagram of studies screened, assessed for eligibility, and included; (**b**) PICO framework, according to the PRISMA checklist [10].

The search was performed based on the PRISMA statement [10]. The following focused question was formulated: “Does the in-vitro application of cold atmospheric plasma show an antimicrobial effect compared to no treatment on pathogens related to dentistry?” According to the PICO criteria defined from the focused question (Figure 1b), a database search was conducted in PubMed (Medline), Embase and Scopus on 19 October 2020 by two authors separately (G.J. and D.M.).

The results were restricted to research articles written in English. The keywords for the PubMed search were: (“cold atmospheric plasma” OR “non-thermal plasma” OR “non-thermal plasma” OR “non-thermal atmospheric plasma” OR “non thermal atmospheric plasma” OR “non-thermal atmospheric pressure plasma” OR “non thermal atmospheric pressure plasma” OR “cold atmospheric pressure plasma” OR “argon plasma” OR “helium plasma” OR “oxygen plasma” OR “nitrogen plasma” OR “air plasma” OR “plasma gases” OR “plasma jet” OR “dielectric barrier discharge” OR “glow discharge” [MeSH Terms]) AND (“dentistry” OR “dental treatment” OR “dental therapy” OR “oral”) AND (“disinfection” OR “sterilization” OR “bacterial inactivation” OR “bactericidal” OR “bacteriostatic” OR “antibacterial” OR “anti-bacterial” OR “microbicidal” OR “antimicrobial” OR “anti-microbial” OR “antifungal” OR “anti-fungal” OR “antiviral” OR “anti-viral”) AND (“in-vitro” OR “in vitro”). For Scopus, the keywords were chosen in the same way and for Embase the PICO manager was used. References from relevant articles were assessed and added when appropriate.

### 2.2. Eligibility Criteria

In-vitro studies investigating the direct application of plasma on pathogens associated with dental diseases were included in the analysis. Therefore, only studies focusing on streptococci, lactobacilli, *E. faecalis, P. gingivalis,* and *A. actinomycetemcomitans* were considered. To compare the outcome only studies specifying the reduction in log_10_ colony forming units (CFU) were included. If the reduction factor (RF) was not outlined, it was calculated using the formula: RF = −log_10_ (CFU_treatment_ /CFU_control_). Results were presented with one decimal place. Reductions less than about 0.5 log_10_ were categorized as non-relevant and declared as ‘no reduction’. Studies not using a control group were excluded. As control no treatment (no Tx), rinsing with sodium chloride (NaCl) or phosphate buffered saline (PBS) or working gas without plasma ignition was accepted. Furthermore, studies investigating the indirect plasma activity on bacteria, i.e., plasma activated water or bacteria in suspension were excluded.

### 2.3. Data Extraction and Assessment of Quality

The data extraction was performed by two authors (G.J. and D.M.) using a piloted form, which included technical parameters of the plasma device, characteristics of the study setup like nozzle-specimen distance, biofilm growth duration, and RF for set treatment time intervals (Table S1). In order to create a comprehensive overview, an additional table was made summarizing RFs for the specific working gases and treatment intervals (Tables 2–5). Disagreements in extracted results were resolved by discussion. To assess the quality of the publications, an assessment tool based on the US EPA protocol and standards for bactericidal activities of disinfectants [11,12,13,14,15] was created. The tool consists of 11 items, nine with one point and two with two points attainable. A maximum score of 13 points in total could be achieved. The final score was expressed as a percentage of achieved points divided by maximum points multiplied by 100. The items evaluated the reproducibility and critical steps within the study protocol that may overestimate the outcome. The detailed description of the assessment tool can be found in Table 1.

### 2.4. Statistical Analysis

The RFs were extracted from tables or read from graphs. The inter-rater reliability for the extracted RFs was calculated using the Intraclass correlation coefficient (ICC; two-way mixed, absolute agreement). ICC < 0.5, 0.5–0.75, 0.75–0.9, >0.9 were interpreted as poor, moderate, good, and excellent reliability respectively [16]. For the quality assessment the inter-rater reliability using Cohen’s kappa coefficient was calculated [17] with the assumption of κ > 0.8/0.6/0.4/0.2/0 as almost perfect, substantial, moderate, fair or slight respectively and κ < 0 as poor agreement [18]. The calculation was performed with SPSS 24 (IBM, Armonk, NY, USA).

## 3. Results

After the duplicates were removed, 2323 articles were screened. Following exclusion by title and abstract, 109 articles could be assessed for eligibility (Figure 1a). Thirty-four studies were excluded because the authors defined another outcome than CFU, four articles did not compare the application of plasma with an untreated control, four treated bacteria in suspension and in five papers the effect on bacterial adhesion and de novo biofilm formation was analyzed. Eight articles did not investigate the antifungal effect related to a dental aspect. The remaining 55 publications were included in the qualitative synthesis (Table 2, Table 3, Table 4 and Table 5). Seventeen articles investigated the antibacterial effect on planktonic bacteria and 37 on bacterial biofilms. One investigated both. Sixteen studies used extracted teeth or a realistic resin tooth model. There, single rooted teeth were decoronated and the root canals were instrumented, before the roots were sterilized, inoculated, and incubated. The inter-rater agreement for the extracted RFs was excellent (ICC: 0.993).

### 3.1. Oral Streptococci

In total, 15 studies exposed streptococci biofilms to plasma, utilizing either different noble gases or ambient air to apply the plasma to the microbes (Table 2). More than half of the studies used an argon plasma device. In the shortest treatment interval of up to 60 s, some groups demonstrated bacterial elimination below the detection level [21,28], whereas others found no significant reduction [19,20,22,25]. For the other time intervals, the RF ranged from no reduction [22] to 3.8 log_10_ CFU [26] within 120 s and from no reduction [22,25] to complete elimination of CFU [24] in 300 s, respectively. Koban et al. applied argon plasma for up to 600 s resulting in a decrease from 1.7 log_10_ CFU for a hollow DBD device to 5.8 log_10_ CFU reduction for a volume DBD [25]. Blumhagen et al. found complete sterilization in 60 s [21]. Molnar et al. used a helium plasma device for 60 and 120 s, respectively. 60 s resulted in a range of 1.3–2.3 log_10_ CFU reduction, 120 s of treatment led to reduction below detection level [32]. For nitrogen plasma, studies with up to 60 and 120 s respectively were performed. In 60 s, values ranged between 0.6 [34] and 1.5 [19], 120 s resulted in 1 log_10_ CFU reduction [34]. Two groups of authors tested DBD plasma devices, utilizing ambient air. RF values for 60 s ranged from 0.6–1.2 log_10_ [31], and for up to 600 s from 2.4–2.6 log_10_ CFU [30]. Rupf et al., who used a combination of helium, nitrous oxide, and oxygen, achieved higher RF values, ranging from 4.0 to 5.8 log_10_ CFU reduction in comparable treatment times [33].

### 3.2. Lactobacilli

In five studies, lactobacilli were treated with different plasma devices (Table 2). Most authors focused on short treatment times of 60 s with devices that used argon as a working gas. In this category, one group of authors found no reduction [19], two others a decrease of CFU below detection level [21,33]. The other two studies demonstrated a consistent but relatively low reduction rate of about 1.0–1.5 log_10_ units. Rupf et al. showed reduction values of 4.5 log_10_ and more using a mixture of helium, nitrous oxide and oxygen [33]. Treating for up to 60 s with nitrous oxide resulted in 0.6–1 log_10_ CFU reduction, pure oxygen rendered values from 1.3 log_10_ to complete sterilization and a combination of both gases showed no reduction up to 0.9 log_10_ CFU [19].

### 3.3. Enterococcus faecalis

In total, 22 studies exposed *E. faecalis* to plasma (Table 3). For the plain model experiments, the carrier gas was either argon or helium, in three studies an admixture of oxygen added to helium was used. Except for the He/O_2_ plasma, the studies demonstrated a higher RF when the treatment time was longer. The reduction values for devices utilizing ambient air in all time intervals were demonstrated by two of the studies. The application of 60 s led to results ranging from no reduction to 7.9 log_10_ reduction units [55]. For pure helium plasma, outcomes ranged from no reduction [45] to no detection of CFU within 60 s [42]. When admixing O_2_, values ranged from 2.9 log_10_ for 10% O_2_, and elimination of all CFU for 2.5% O_2_ in up to 60 s treatment [43].

Six groups of authors utilized argon or Ar/O_2_ plasma, eight helium or He/O_2_ plasma on *E. faecalis* cultures inoculated in root canals of extracted teeth. In one study an air-driven jet was used additionally [52]. Ballout et al. used a DBD device and showed no significant reduction after an application time of 60 s [35]. Concerning plasma devices operating with pure argon, the reduction ranged from no reduction [35] to 3.2 log_10_ units [37] for treatment up to 60 s. A treatment time over 10 minutes led to a reduction of 1.8 log_10_ CFU in minimum [36] and to a non-detectable number in maximum [39]. For devices operating with argon plus an admixture of oxygen, values ranged from no reduction to 6.6 log_10_ CFU for up to 120 s [38,40] Eight studies utilizing helium or helium plus admixtures were included. For pure helium, no reduction in the time less or equal 60 s was shown [45]. In the same study, the reduction in the group up to 300 s was 4.0 log_10_ [45], whereas another group observed no reduction [44]. Higher RFs were achieved when admixing O_2_ to helium. For the interval up to 300 s the reduction ranged from 2.8 [47] to 5.1 log_10_ CFU [44]. A Chinese group investigated different admixtures to helium and He/O_2_ gas. They let the working gas flow through hydrogen peroxide (H_2_O_2_) [52] and sodium hypochloride (NaOCl) [51] before the plasma was ignited. The values of the RF were: 4.9 log_10_ (He plus H_2_O_2_) in up to 60 s, 7.0 log_10_ (He plus H_2_O_2_) and 2.2 log_10_ (He/O_2_ plus NaOCl) in up to 300 s, 2.4 log_10_ (He/O_2_ plus NaOCl) in up to 600 s and 5.4 log_10_ CFU for a treatment time over 600 s.

### 3.4. Periopathogens

Three studies report on the activity of plasma on *P. gingivalis* (Table 4). For *P. gingivalis*, the maximum reduction in the time interval up to 60 s was 1.2 log_10_ CFU for argon [57]. Up to 120 s air plasma reached 4.0 log_10_ CFU [58]. In the group up to 300 s a RF of 1.2 log_10_ CFU was achieved for argon [57] and helium plasma [59]. In the same interval, the air driven device obtained a reduction of 4.5 log_10_ CFU [58].

Three studies investigated the effect of CAP on *A. actinomycetemcomitans* (Table 4). Direct application of argon plasma showed a non-significant reduction compared to the control group at a treatment time of less than 1 minute [19], but the numbers of CFU decreased to a non-detectable level after more than 10 minutes [56]. When using different working gases, the values of RF increased. For oxygen plasma, a reduction of 1.9 log_10_ CFU and a reduction below the detection level was shown for up to 60 s at treatment distances of 20 and 2 mm, respectively [19]. Using a DBD values for less than 1 min treatment time range from 1.1 to 2.2 log_10_ and for 2 min from 1.8 to 2.8 log_10_ CFU reduction [31]. Consistently, a time-dependent increase of the bacterial inactivation was evident among the studies.

### 3.5. Candida

Fourteen studies investigated the antifungal properties of CAP on *C. albicans* and *Candida krusei* (Table 5). Using pure Ar plasma, the RF ranged from no reduction for a jet device to 2.3 log_10_ CFU for volume DBD within a treatment time up to 60 s, and from 0.5 for jet and 2.2 log_10_ up to 120 s [73]. For the time interval up to 300 s, the reductions were between no deactivation [64] and 3.5 log_10_ CFU [73]. Highly matured biofilm, grown for 7 and 16 days, showed no reduction after a treatment time of up to 600 s [61], whereas the values for 24–48 hours biofilm ranged from no reduction to 5.2 log_10_ CFU dependent on the device type [73]. Admixing O_2_ to Ar the attained reduction was very similar compared to pure Ar. Kerlikowski et al. used an Ar/O_2_ jet device to treat *C. albicans* in root canals. The reduction achieved was 2.1 CFU in less than 600 s [65]. He/O_2_ resulted in total inactivation of a *C. albicans* and a *C. krusei* biofilm within 60 s [72], whereas another group of authors showed a reduction of 0.4 to 0.8 log_10_ CFU dependent on the amount of oxygen admixed [70]. Using an air-based device, Maisch et al. attained a reduction of 5.5 log_10_ in 60 s and a total elimination in all longer application intervals when treating planktonic *C. albicans*. For a 24-hours biofilm, the RF was 0.7 log_10_ after 120 s of treatment.

### 3.6. Multi-Species Biofilm

Two studies were performed investigating the effect on an ex-vivo, multi-species biofilm. The first one was published by Koban et al. analyzing the inactivation ability of different devices and oxygen admixtures on an aerobically cultured saliva biofilm. The reduction values for a pure argon jet were 1.6, 1.8, 1.5, and 1.4 log_10_ for treatment intervals of up to 60, 120, 300, and 600 s, respectively. When adding oxygen, no reduction was found at any time interval. For a hollow DBD device values were 1.2, 1.6, 1.4, and 1.5 log_10_. An oxygen admixture resulted in no reduction for 60 s, and 1.4, 3.1, 2.2 log_10_ for 120, 300, and 600 s. The highest reduction was obtained using a volume DBD device showing 3.9, 3.7, 5.3, and 5.6 log_10_ reduction in up to 60, 120, 300, and 600 s treatment, respectively [25]. Secondly, one study was conducted treating an ex-vivo biofilm in a root canal. The obtained reduction was 1.0 log_10_ CFU for an application time of 30 min in total [48].

### 3.7. Quality Assessment

The scores in the quality assessment ranged from 38% to 92% while 54% (11/55 publications), 62% (10/55 publications), and 77% (10/55 publications) were achieved most frequently (Table S2). Eight articles were rated under 50%. The inter-rater agreement was almost perfect (κ = 0.825). A point in item 5 was assigned when measures are described to protect specimens in surrounding wells, when not actively treated, to avoid cumulative effects caused by generated ozone and dehydration, for example. In two out of 55, a point for item 5 was obtained [60,69].

## 4. Discussion

This systematic review intended to give an overview of the current in-vitro data concerning the antimicrobial effect of CAP on pathogens associated with dental diseases. Furthermore, factors which should be considered for further in-vitro studies and for future clinical applications were defined. Due to the high heterogeneity of the study designs—e.g., device parameters like power, frequency, working gas composition, application time, nozzle-specimen distance, underlying surface, and biofilm growth duration, that influence the outcome—a quantitative synthesis was not considered to be beneficial. In the majority of the studies, a reduction of 3 or more log_10_ units in vitro, which is considered to be bactericidal [74], was achieved within a treatment time of 2 minutes. The antibacterial activity increased in a time-dependent manner. This suggests that the application of CAP may represent a promising alternative or an adjunctive in antibacterial therapy.

Thirteen studies were conducted using a commercially available device. In 12 studies kINPen (neoplas med, Greifswald, Germany) was used, and in two of these 12 studies additionally PlasmaDerm (CYNOGY System, Duderstadt, Germany). One study tested Plasma R (Sweden & Martina, Due Carrare, Italy). Forty-two groups of authors tested self-constructed devices. Various device parameters influenced the outcome and should therefore be taken into account in terms of reproducibility. An increase in power [28,42] enhanced the bacterial inactivation, but also results in an increase of plasma temperature [42]. Additionally, the gas flow influences the plasma temperature [75]. To avoid thermal damage of the treated tissues the device parameters need to be adjusted [76]. Some devices used in the included studies are not designed for a chairside usage, e.g., plasma was generated in a locked box filled with noble gas [56,67]. These studies rather proved the principle. For an implementation in clinical practice, the devices need to be designed as a hand-held device and a plasma generation with ambient air is favorable over pressurized gas cylinders.

The question, whether adding oxygen to a noble gas plasma device results in better RF values, remains unclear based on the included studies. This may be due to the fact that different types of devices were used and the advantageous effect of O_2_ admixture depends on the mode of plasma generation [25]. When adding oxygen near the grounded electrode of a DBD device, the reduction of CFU did not differ compared to pure argon [21]. For a DBD brush device, Chen et al. showed that adding 1.0 to 2.5% of oxygen to helium plasma was favorable in reducing *E. faecalis* biofilms. The admixture of 5% oxygen to helium also showed an increased antifungal effect on a *C. albicans* biofilm [70]. The antimicrobial properties of CAP are attributed to the generation of ROS, RNS, electrons, and UV radiation, whereas ROS is supposed to play the main part [3,77]. In the electric field between the two electrodes of the plasma device, the atoms of the noble gases are ionized, and electrons are released. These electrons react with oxygen and nitrogen and form radicals. In terms of a brush device, adding oxygen up to 2.5–5% of volume prior to the working gas ionization results in an increased microbicidal inactivation due to an elevated ROS generation. However, using higher percentages of oxygen had a contrary effect [43,70]. Song et al. explained that by a higher density of reactive species which results in augmented collisions with free electrons within the electric field and a lesser number of radicals in the effluent [70].

Admixture of hydrogen peroxide 3% to helium also enhances the bacterial deactivation by enhancing ROS production as analyzed in the optical emission spectrum [52].

The values of RF varied markedly among the studies although a cluster within the ranges could be seen. Various devices were used which affect the outcome as described earlier. However, differences in specimen preparation, biofilm growth duration and bacterial characteristics also affected the results. To evaluate the possible indication of CAP application in the various fields of dentistry bacteria were grown on different surfaces like dentine or hydroxyapatite for cariogenic, titanium for peri-implant biofilms, or porcine bone for osteitis. Some studies simply used laboratory consumables like well-plates, agar in Petri dishes, cover glasses, or filter paper. Taken together the findings of the plain surface experiments, a bigger roughness of porous surfaces offers a cover to evade the plasma assault [20,26,33]. The achieved reduction in log_10_ CFU was also dependent on the inoculum size. Lower concentrations of *S. mutans* and *L. acidophilus* resulted in an increased inactivation [21,28,32]. In general, biofilm maturation was associated with an augmented resistance to CAP application in Gram-positive [22,24,31,55] and Gram-negative species [31]. The maturation of the biofilm is accompanied with the production of an extracellular matrix and an increase of biofilm thickness. Therefore, the deeper cell layers are better protected and need an extended treatment time for inactivation [24]. Fifteen studies were performed using a single-species *S. mutans* biofilm. This Gram-positive coccus is the most relevant pathogen in the establishment of carious lesions and therefore commonly used to investigate dental biofilms, but it is also recently advancing to a model organism for biofilm in microbiology due to its facilitated handling [78]. In terms of a caries preventive therapy, Sprague-Dawley rats were treated with CAP for 2 min on the molars of one side, the other side served as control. After six months the caries rate on the test side was decreased by 20% compared to the control [29]. Another possible clinical application could be the disinfection of the cavity after caries excavation. CAP eliminates remaining pathogens and alters the tooth surface. CAP treatment showed an increase in bonding strength in the dentin–adhesive interface [79,80,81] and enhanced enamel remineralization [82,83]. This may prevent secondary caries.

In an inhospitable environment like the oral cavity, microorganisms were forced to develop surviving strategies. Therefore, they organize themselves in a symbiotic community supporting each other [5]. Multi-species biofilms are less susceptible to chemical antimicrobial agents, antibiotics [84], and to CAP [85]. In the study of Koban et al., the RF for *S. mutans* biofilm was 3.1, whereas the RF for an ex-vivo, saliva biofilm was 1.6 log_10_ CFU using an argon plasma jet for 60 s [25]. Another aspect of the latter publication should be taken into account when interpreting the results. The *S. mutans* and the saliva biofilms were incubated aerobically. The authors stated that this may result in a selection of more aerotolerant species within the saliva biofilm and a suppression of Gram-negative anaerobic species, which are more susceptible to ROS [25]. Therefore, the lower antibacterial effect can be caused by the multi-species biofilm or by the pre-treatment selection due to the aerobic incubation. Furthermore, the altered biofilm composition may not represent the clinical reality.

Six studies elucidated the antibacterial effect on Gram-negative periopathogens *P. gingivalis* and *A. actinomycetemcomitans*. The effect of CAP on other microorganisms associated with periodontal disease [8] was not analyzed. Within the limitations of this review both periopathogens tend to be more susceptible to plasma treatment than the Gram-positive species. A higher susceptibility of Gram-negative organisms compared to Gram-positives is consistent with recent reports on non-oral species [86,87]. Yang et al. used ambient air as working gas. They showed a higher RF when compared with pure argon- [57] or pure helium-driven devices [59]. *P. gingivalis* was the only obligate anaerobic pathogen in the studies included and therefore maybe more sensitive to an elevated amount of ROS induced by plasma, although *P. gingivalis* developed various strategies to resist oxidative stress [88]. Bacterial decontamination is crucial in periodontal and peri-implant therapy. The macro- and micro-retentive surface of dental implants is challenging to decontaminate. Duske et al. treated subgingival biofilm on sand-blasted and etched titanium discs. The SEM images showed that only a combined treatment with a titanium brush and CAP resulted in an adequate decontamination [89]. In the combination of air abrasion and CAP, CAP did not result in additional effect. In this study, air abrasion by itself resulted in complete biofilm elimination. These tests were performed with an optimal access to the treated surface [90]. The activity of different decontamination methods in correlation to the defect angulation was further investigated in vitro. The authors showed for all instruments a decrease in decontamination depending on the steepness of the defect and no instrument achieved complete decontamination in steep defects [91]. Therefore, in a clinical setting, an adjunctive antiseptic together with a mechanical decontamination might be beneficial as shown in the literature [92]. CAP application may be a promising alternative to the current adjunctives. Additional application of CAP resulted in significant higher peri-implant bone level and less inflammation compared to conventional treatment with plastic curettes in a ligature-induced peri-implant disease model in beagle dogs 3 months after the treatment. Furthermore, levels of *P. gingivalis* and *Tannerella forsythia* were significantly decreased compared to the control [93]. In the only RCT published, patients were treated with adjunctive CAP and showed significant more attachment level gain in severe periodontal pockets and a reduced load of periopathogens compared to conventional treatment 3 months after therapy [94].

The antifungal effect of CAP on *C. albicans* was investigated in 14 studies, 13 on plain surfaces, and 1 in an endodontic model [65]. In accordance with the findings from the bacterial experiments, the reduction increased with in a time-dependent manner. Adding oxygen had an advantageous effect when using a DBD brush device [70], equivalent to the experiments done by Chen et al. [43], but also when using a jet device [73]. The reduction values in the included studies seemed to be lower than for bacterial species. This may be to a higher resistance of the aerobe yeasts to CAP-generated ROS [95]. Biofilm maturation resulted in significant lower reductions [61,67].

Üreyen et al. demonstrated a RF of 3.1 log_10_, when sampling the bacteria with paper points after treatment with He/O_2_ plasma for 5 min. Additionally, they separated the roots in thirds, enlarged the canals with burs and recovered the bacteria from the gained dentin chips. The values for the coronal, middle and apical third were 3.2 log_10_, below detection level and 3.4 log_10_, respectively. This shows that the antibacterial effect can be substantiated over the total length of the root canal [50]. *E. faecalis* is capable of penetrating the radial dentin tubuli up to 1000 µm [7] and may reinfect the filled root canal system after the primary treatment. It is a predominant pathogen in secondary endodontic infections due to its increased resistance against antiseptics [96]. Herbst et al. additionally analyzed the penetration depth effect of plasma. The RF was 3.4, 2.1, and 1.4 log_10_ for the perpendicular dentin sections of 0–300 µm, 300–500 µm, and 500–800 µm, respectively [37]. For NaOCl, penetration depths of approximately 100–300 µm in maximum—when activated—are described in the literature [97,98,99,100]. Herbst et al. showed a higher RF for CHX 2% compared to CAP in the 500–800 µm layer [37]. However, there were also contrary reports in regard to the standard irrigants used for endodontic treatment. CAP showed a similar [47] or even slightly better reduction on *E. faecalis* biofilms compared to CHX 2% [37,39], and a similar [38] or lower RF compared to NaOCl [35,48,50].

When treating a *C. albicans* biofilm, CAP showed a better antifungal effect compared to CHX 2% and NaOCl in a 6 and 12 min application time [65]. Against a multi-species, ex-vivo biofilm NaOCl was far more effective than CAP resulting in a reduction of 4.5 log_10_ compared to 1.0 log_10_, respectively. The authors argue that even when inserting the plasma needle up to 15 mm into the root canal the plasma effluent is unable to interact over a longer distance [48]. Contrary findings were made by Du et al. showing no significant difference in the reduction between straight and complex canal anatomies even when inserting plasma nozzle only into the canal orifice [47]. Another interesting aspect of plasma application is the reduction of the surface tension [101]. An additional advantageous effect on the irrigants’ disinfecting capability [65,102] and an improved adhesion of the restorative material, especially after NaOCl rinsing [77,103], makes CAP a promising extension to the established endodontic treatment protocols. In terms of oral candidiasis, BALB/c mice were infected with *C. albicans* and treated with CAP 5 days consecutively. Four minutes of treatment appeared ideal for adequate reduction and no damage of the superficial epithelium [71].

An assessment tool was designed to evaluate the reproducibility of the study protocols. The quality of the studies ranged from 32–92%. Less than half of the studies (23 of 55) had a quality score of 69% or higher. In nine studies the protocol was described so insufficiently that the quality score was under 50%. When authors fail to give relevant parameters, the experiments can hardly be repeated and compared. A limitation of this article is that due to the heterogeneous results by reason of device parameters and experimental settings a meta-analysis could not be performed. To access appropriate publications referring to our study question we performed a database search and only included research articles. Generally, studies with a high effect of a new treatment option may be published in peer-reviewed journals more often than studies showing a small impact of a treatment. Some results of the studies included in our review were not significantly different to the control, therefore an overestimated effect of CAP due to a publication bias is rather improbable.

## 5. Conclusions

The available evidence from in-vitro studies suggests that CAP is a promising tool in combating dental biofilms. Significant reductions can be achieved in a feasible treatment time, although the current data showed a broad range of values. Underlying mechanisms and specific plasma microbe interactions are not fully understood yet and discussed controversially in the literature. Additional studies are needed to enlighten the correlation of power and oxygen admixture with regards to the type of device. Furthermore, brush devices igniting plasma in ambient air seem to be auspicious because no gas tanks are necessary and therefore the clinical implementation of CAP might be easier. To compare the activity of different devices, the experimental set-up needs to be standardized to reduce variations in outcome determining factors like specimen preparation, inoculum size, growth duration of the biofilm, and treatment times. Most studies analyze the antimicrobial effect on single-species biofilms. Multi-species biofilms are more challenging and clinically more relevant. More studies using predetermined biofilm compositions need to be done. Finally, animal and clinical studies are required to confirm the results found in these in-vitro experiments. Possible indications in cariology, endodontology, periodontology, and implantology are conceivable.

## Figures and Tables

**Table 1:** Quality assessment tool.

Critical Steps of the in Vitro Protocol	Justification	Items	Points Attributed According to the Response for Each Critical Step
Preparation of micro-organisms and plasma device	Scientific robustness	1. Preparation of microorganisms	1 if described 0 if not described
		2. Technical data of plasma generator	2 if at least 3 parameters described or commercially device 1 if at least 1 parameter described 0 if not described
Inoculation (Inoculum size)	Real inoculum size essential for the calculation of logarithmic reduction	3. Experimental size presented	2 for theoretical + true inoculum sizes 1 for theoretical inoculum size 0 if not described
Test conditions	Scientific robustness	4. Experimental temperature	1 if described 0 if not described or over 47 °C
		5. Protection of samples	1 if described 0 if not described
Micro-organisms recovery	Impacts the results if not all micro-organisms are recovered, overestimated effect Scientific robustness	6. Micro-organisms recovery	1 if other method with mechanic action and validated with a test 0 if not clearly described or technic not validated
Microbial culture after treatment	Impacts the results if the growth duration is too short Scientific robustness	7. Time, temperature and method indicated	1 if described 0 if not or poorly described
		8. Culture media	1 if described 0 if not described
Statistical analysis/tests repeatability	Scientific robustness	9. Number of experiments	1 if described with more than one experiment 0 if not described or described with onlyone experiment
		10. Statistical method (to compare differences)	1 if described 0 if not described
Conflict of Interest	Bias	11. Declaration	1 if declared 0 if not declared

The global score was calculated by summing each point (score = sum/13*100).

**Table 2:** Effect of CAP on bacteria associated with dental caries; Abbreviations: CD: corona discharge; conc.: concentration; DBD: dielectric barrier discharge; HA: hydroxyapatite; HDBD: hollow dielectric barrier discharge; max red: maximal reduction; med.: medium; pos.: positive; PTFE: polytetrafluorethylene; N/A: not available; nd: non-detectable; neg.: negative; ns: non-significant; self-constr.: self-constructed; Ti: titanium; VDBD: volume dielectric barrier discharge.

Gas	Author	Device	Plasma Mode	Distance (mm)	Surface	Biofilm	Species	Max Red t ≤ 60 s	Max Red t ≤ 120 s	Max Red t ≤ 300 s	Max Red t ≤ 600 s	Max Red t > 600 s	Additional
*Ar*	Abonti et al. 2016 [19]	self-constr.	jet	2	Agar	No	*S. mutans*	ns (1.4)	N/A	N/A	N/A	N/A	
		self-constr.	jet	20	Agar	No	*S. mutans*	ns (1.6)	N/A	N/A	N/A	N/A	
		self-constr.	jet	2	Agar	No	*L. fermentum*	ns (1.1)	N/A	N/A	N/A	N/A	
		self-constr.	jet	20	Agar	No	*L. fermentum*	ns (0.7)	N/A	N/A	N/A	N/A	
	Abu-Sirhan et al. 2016 [20]	kINPen med	jet	8	bone (porcine)	Yes	*S. mitis*	ns (0.2)	N/A	N/A	N/A	N/A	
	Blumhagen et al. 2014 [21]	self-constr.	brush	5	HA discs	No	*L. acidophilus*	1.7	N/A	N/A	N/A	N/A	high conc.
		self-constr.	brush	5	HA discs	No	*L. acidophilus*	nd	N/A	N/A	N/A	N/A	med. conc., nd after 13 s
		self-constr.	brush	5	HA discs	No	*L. acidophilus*	nd	N/A	N/A	N/A	N/A	low conc., nd after 10 s
		self-constr.	brush	5	HA discs	No	*L. acidophilus*	nd	N/A	N/A	N/A	N/A	nd after 13 s
		self-constr.	brush	5	HA discs	No	*L. acidophilus*	nd	N/A	N/A	N/A	N/A	nd after 13 s
		self-constr.	brush	5	HA discs	No	*L. acidophilus*	nd	N/A	N/A	N/A	N/A	nd after 13 s
		self-constr.	brush	5	HA discs	No	*S. mutans*	2.6	N/A	N/A	N/A	N/A	high conc.
		self-constr.	brush	5	HA discs	No	*S. mutans*	nd	N/A	N/A	N/A	N/A	med. conc., nd after 13 s
		self-constr.	brush	5	HA discs	No	*S. mutans*	nd	N/A	N/A	N/A	N/A	low conc., nd after 6 s
		self-constr.	brush	5	HA discs	No	*S. mutans*	nd	N/A	N/A	N/A	N/A	nd after 13 s
		self-constr.	brush	5	HA discs	No	*S. mutans*	nd	N/A	N/A	N/A	N/A	nd after 13 s
		self-constr.	brush	5	HA discs	No	*S. mutans*	nd	N/A	N/A	N/A	N/A	nd after 17 s
	Gorynia et al. 2013 [22]	kinpen 09	jet	10	Ti discs	Yes	*S. sanguinis*	ns (0.1)	ns (0.3)	nd (0.5)	N/A	N/A	
	Hertel et al. 2018 [23]	kINPen med	jet	8	Dentin	Yes	*L. rhamnosus*	1.1	N/A	N/A	N/A	N/A	
		PlasmaDerm	VDBD	close contact	Dentin	Yes	*L. rhamnosus*	0.6	N/A	N/A	N/A	N/A	
	Huang et al. 2013 [24]	self-constr.	jet	3	Glass	No	*S. mutans*	2.7	3.1	nd	N/A	N/A	
		self-constr.	jet	3	Glass	No	*S. mutans*	1.5	2.5	4.8	N/A	N/A	
	Koban et al. 2011 [25]	kINPen 09	jet	7	Ti discs	Yes	*S. mutans*	3.1	2.8	3	3	N/A	
			HDBD	5	Ti discs	Yes	*S. mutans*	1	1.2	1.7	1.7	N/A	
			VDBD	15	Ti discs	Yes	*S. mutans*	2	3.3	5.1	5.8	N/A	
	Park et al. 2014 [26]	self-constr.	jet	8	Glass	No	*S. mutans*	3	3.8	5.3	N/A	N/A	
		self-constr.	jet	8	Tooth	No	*S. mutans*	3	3	3.4	N/A	N/A	
	Preissner et al. 2016 [27]	kINPen MED	jet	8	Ti implants	Yes	*S. mitis*	2.2	1.9	N/A	N/A	N/A	
	Yang et al. 2011 [28]	self-constr.	brush		Filter paper	Yes	*S. mutans*	nd	N/A	N/A	N/A	N/A	nd after 11 s
		self-constr.	brush		Filter paper	Yes	*S. mutans*	nd	N/A	N/A	N/A	N/A	nd after 9 s
		self-constr.	brush		Filter paper	Yes	*S. mutans*	nd	N/A	N/A	N/A	N/A	nd after 15 s
		self-constr.	brush		Glass slide	Yes	*S. mutans*	nd	N/A	N/A	N/A	N/A	nd after 15 s
		self-constr.	brush		PTFE	Yes	*S. mutans*	nd	N/A	N/A	N/A	N/A	nd after 13 s
		self-constr.	brush		PTFE	Yes	*S. mutans*	nd	N/A	N/A	N/A	N/A	10% density, nd after 5 s
		self-constr.	brush		PTFE	Yes	*S. mutans*	nd	N/A	N/A	N/A	N/A	1% density, nd after 3 s
		self-constr.	brush		Filter paper	Yes	*L. acidophilus*	2.2	3.9	nd	N/A	N/A	
		self-constr.	brush		Filter paper	Yes	*L. acidophilus*	3.7	4.3	nd	N/A	N/A	nd after 210 s
		self-constr.	brush		Filter paper	Yes	*L. acidophilus*	1.9	3.2	nd	N/A	N/A	nd after 300 s
		self-constr.	brush		Glass slide	Yes	*L. acidophilus*	1.4	2.4	nd	N/A	N/A	
		self-constr.	brush		PTFE	Yes	*L. acidophilus*	2	3	nd	N/A	N/A	
		self-constr.	brush		PTFE	Yes	*L. acidophilus*	3.9	nd	N/A	N/A	N/A	10% density, nd after 90 s
		self-constr.	brush		PTFE	Yes	*L. acidophilus*	nd	N/A	N/A	N/A	N/A	1% density
*Ar + O_2_*	Blumhagen et al. 2014 [21]	self-constr.	brush	5	HA discs	Yes	*L. acidophilus*	nd	N/A	N/A	N/A	N/A	Ar/O_2_: 60:1, nd after 13 s
		self-constr.	brush	5	HA discs	Yes	*L. acidophilus*	nd	N/A	N/A	N/A	N/A	Ar/O_2_: 6:1, nd after 13 s
		self-constr.	brush	5	HA discs	Yes	*L. acidophilus*	nd	N/A	N/A	N/A	N/A	Ar/O_2_: 1:2, nd after 13 s
		self-constr.	brush	5	HA discs	Yes	*S. mutans*	nd	N/A	N/A	N/A	N/A	Ar/O_2_: 300:5, nd after 13 s
		self-constr.	brush	5	HA discs	Yes	*S. mutans*	nd	N/A	N/A	N/A	N/A	Ar/O_2_: 6:1, nd after 13 s
		self-constr.	brush	5	HA discs	Yes	*S. mutans*	nd	N/A	N/A	N/A	N/A	Ar/O_2_: 1:2, nd after 17 s
	Hong et al. 2019 [29]	self-constr.	brush		Steel wafers	Yes	*S. mutans*	0.3	0.5	N/A	N/A	N/A	Ar/O_2_: 100:1
	Koban et al. 2011 [25]	kINPen 09	jet	7	Ti discs	Yes	Saliva	ns (0.3)	ns (0.5)	ns (0.4)	N/A	ns (0.7)	Ar/O_2_: 100:1
		kINPen 09	jet	7	Ti discs	Yes	*S. mutans*	2	1.9	2.1	N/A	1.8	Ar/O_2_: 100:1
			HDBD	5	Ti discs	Yes	Saliva	ns (0.5)	1.4	3.1	N/A	2.2	Ar/O_2_: 100:1
			HDBD	5	Ti discs	Yes	*S. mutans*	ns (0)	0.9	3	N/A	3.7	Ar/O_2_: 100:1
*Air*	Hertel et al. 2018 [23]	PlasmaDerm	VDBD	0	Dentin	Yes	*L. rhamnosus*	0.6	N/A	N/A	N/A	N/A	
	Kovalova et al. 2014 [30]	self-constr.	pos. CD	5	Teeth	Yes	*Streptococci*	N/A	0.9	1	2.6	N/A	
		self-constr.	neg. CD	5	Teeth	Yes	*Streptococci*	N/A	0.8	1.3	2.4	N/A	
	Liguori et al. 2017 [31]	self-constr.	DBD-Rod	3	Well Plate	No	*S. mutans*	2.1	2.9	N/A	N/A	N/A	
		self-constr.	DBD-Plate	3	Well Plate	No	*S. mutans*	1.8	2.8	N/A	N/A	N/A	
		self-constr.	DBD-Rod	3	Well Plate	Yes	*S. mutans*	0.8	1.8	N/A	N/A	N/A	
		self-constr.	DBD-Plate	3	Well Plate	Yes	*S. mutans*	0.6	1.6	N/A	N/A	N/A	
*He*	Molnar et al. 2013 [32]	self-constr.	DBD	2	Tooth Slices	Yes	*S. mutans*	1.3	nd	N/A	N/A	N/A	high conc.
		self-constr.	DBD	2	Tooth Slices	Yes	*S. mutans*	2.4	nd	N/A	N/A	N/A	med. conc.
		self-constr.	DBD	2	Tooth Slices	Yes	*S. mutans*	2.3	nd	N/A	N/A	N/A	low conc.
*He + O_2_ + N_2_*	Rupf et al. 2010 [33]	self-constr.	jet	1.5	Agar	Yes	*S. mutans*	5.8	8.4	N/A	N/A	N/A	He/O_2_/N_2_: 2:1.2:1.5
		self-constr.	jet	1.5	Dentin slice	No	*S. mutans*	4	N/A	N/A	N/A	N/A	He/O_2_/N_2_: 2:1.2:1.5
		self-constr.	jet	1.5	Agar	Yes	*L. casei*	nd	nd	N/A	N/A	N/A	He/O_2_/N_2_: 2:1.2:1.5
		self-constr.	jet	1.5	Dentin slice	No	*L. casei*	4.5	N/A	N/A	N/A	N/A	He/O_2_/N_2_: 2:1.2:1.5
*N_2_*	Abonti et al. 2016 [19]	self-constr.	jet	2	Agar	No	*S. mutans*	6.2	N/A	N/A	N/A	N/A	
		self-constr.	jet	20	Agar	No	*S. mutans*	7	N/A	N/A	N/A	N/A	
		self-constr.	jet	2	Agar	No	*L. fermentum*	1.8	N/A	N/A	N/A	N/A	
		self-constr.	jet	20	Agar	No	*L. fermentum*	2.3	N/A	N/A	N/A	N/A	
	Yoo et al. 2020 [34]	self-constr.	jet		HA discs	Yes	*S. mutans*	0.6	1	N/A	N/A	N/A	
*O_2_*	Abonti et al. 2016 [19]	self-constr.	jet	2	Agar	No	*S. mutans*	nd	N/A	N/A	N/A	N/A	
		self-constr.	jet	20	Agar	No	*S. mutans*	nd	N/A	N/A	N/A	N/A	
		self-constr.	jet	2	Agar	No	*L. fermentum*	2.4	N/A	N/A	N/A	N/A	
		self-constr.	jet	20	Agar	No	*L. fermentum*	nd	N/A	N/A	N/A	N/A	
*O_2_ + N_2_*	Abonti et al. 2016 [19]	self-constr.	jet	2	Agar	No	*S. mutans*	5.2	N/A	N/A	N/A	N/A	
		self-constr.	jet	20	Agar	No	*S. mutans*	6.8	N/A	N/A	N/A	N/A	
		self-constr.	jet	2	Agar	No	*L. fermentum*	ns (1.7)	N/A	N/A	N/A	N/A	
		self-constr.	jet	20	Agar	No	*L. fermentum*	2.3	N/A	N/A	N/A	N/A	

**Table 3:** Effect of CAP on *E. faecalis*; Abbreviations: conc.: concentration; DBD: dielectric barrier discharge; HA: hydroxyapatite; H_2_O_2_: hydroxy peroxide; max red: maximal reduction; N/A: not available; NaOCl: sodium hypochlorite; nd: non-detectable; ns: non-significant; self-constr.: self-constructed.

Gas	Author	Device	Plasma mode	Distance (mm)	Surface	Biofilm	Species	Max Red t ≤ 60 s	Max Red t ≤ 120 s	Max Red t ≤ 300 s	Max Red t ≤ 600 s	Max Red t > 600 s	Additional
*Ar*	Ballout et al. 2018 [35]	kINPen med	jet	3	Root canals	Yes	*E. faecalis*	0.5	N/A	N/A	N/A	N/A	
	Hüfner et al. 2017 [36]	kINPen 08	jet	3	Root canals	Yes	*E. faecalis*	N/A	N/A	N/A	1.5	1.8	
	Herbst et al. 2015 [37]	kINPen MED	jet	3	Root canals	Yes	*E. faecalis*	3.2	N/A	N/A	N/A	N/A	
*Ar + O_2_*	Hüfner et al. 2017 [36]	kINPen 08	jet	3	Root canals	Yes	*E. faecalis*	N/A	N/A	N/A	0.5	1.9	Ar/O_2_: 100:1
	Habib et al. 2014 [38]	self-constr.	jet		Root canals	Yes	*E. faecalis*	N/A	6.6	N/A	N/A	N/A	Ar/O_2_: 1000:1
	Li et al. 2015 [39]	self-constr.	jet	10	Root canals	Yes	*E. faecalis*	N/A	N/A	2.3	6.3	nd	Ar/O_2_: 98:2
	Pan et al. 2013 [40]	self-constr.	jet	5	Root canals	Yes	*E. faecalis*	N/A	0.6	1.2	nd	N/A	Ar/O_2_: 98:2
	Wang et al. 2011 [41]	self-constr.	jet	5	Root canals	No	*E. faecalis*	N/A	0.2	0.8	2	N/A	Ar/O_2_
*He*	Chen et al. 2012 [42]	self-constr.	brush	5	Filter paper	Yes	*E. faecalis*	2.7	3	4.5	N/A	N/A	16 W
		self-constr.	brush	5	Filter paper	Yes	*E. faecalis*	3.4	4	nd	N/A	N/A	20 W
		self-constr.	brush	5	Filter paper	Yes	*E. faecalis*	4	nd	nd	N/A	N/A	24 W
		self-constr.	brush	5	Filter paper	Yes	*E. faecalis*	5	nd	nd	N/A	N/A	28 W
		self-constr.	brush	5	Filter paper	Yes	*E. faecalis*	nd	nd	nd	N/A	N/A	32 W
	Chen et al. 2012 [43]	self-constr.	brush	5	Filter paper		*E. faecalis*	4	nd	nd	N/A	N/A	
	Armand et al. 2019 [44]	self-constr.	jet	2	Root canals	Yes	*E. faecalis*	N/A	N/A	1	2.1	N/A	
	Simoncelli et al. 2015 [45]	self-constr.	jet	2	Root canals	No	*E. faecalis*	0.2	N/A	4	N/A	N/A	
*He + O_2_*	Jiang et al. 2012 [46]	self-constr.	jet	10	HA discs	Yes	*E. faecalis*	N/A	N/A	1.2	N/A	N/A	He/O_2_: 100:1
	Chen et al. 2012 [43]	self-constr.	brush	5	Filter paper		*E. faecalis*	4.8	nd	nd	N/A	N/A	He + 1% O_2_
		self-constr.	brush	5	Filter paper		*E. faecalis*	nd	nd	nd	N/A	N/A	He + 2.5% O_2_
		self-constr.	brush	5	Filter paper		*E. faecalis*	3.7	5.2	nd	N/A	N/A	He+ 5% O_2_
		self-constr.	brush	5	Filter paper		*E. faecalis*	2.9	3	3.7	N/A	N/A	He + 10% O_2_
	Armand et al. 2019 [44]	self-constr.	jet	2	Root canals	Yes	*E. faecalis*	N/A	N/A	1.4	5.1	N/A	He/O_2_: 200:1
	Du et al. 2012 [47]	self-constr.	jet	5	Root canals	Yes	*E. faecalis*	N/A	N/A	0.9	2.8	3.2	He/O_2_: 100:1
	Schaudinn et al. 2013 [48]	self-constr.	jet		Root canals	Yes	ex vivo	N/A	N/A	N/A	N/A	1	He/O_2_: 99:1
	Lu et al. 2009 [49]	self-constr.	jet	2	Root canals	Yes	*E. faecalis*	N/A	N/A	N/A	N/A	2	He/O_2_: 80:20
	Üreyen et al. 2014 [50]	self-constr.	DBD	−1	Root canals	Yes	*E. faecalis*	N/A	N/A	3.1	N/A	N/A	He/O_2_: 96:4
		self-constr.	DBD	−1	Root canals	Yes	*E. faecalis*	N/A	N/A	3.2	N/A	N/A	He/O_2_: 96:4
		self-constr.	DBD	−1	Root canals	Yes	*E. faecalis*	N/A	N/A	nd	N/A	N/A	He/O_2_: 96:4
		self-constr.	DBD	−1	Root canals	Yes	*E. faecalis*	N/A	N/A	3.4	N/A	N/A	He/O_2_: 96:4
	Zhou et al. 2010 [51]	self-constr.	jet	0	Root canals	Yes	*E. faecalis*	N/A	N/A	1.7	2.2	4.5	He/O_2_: 100:1
		self-constr.	jet	0	Root canals	Yes	*E. faecalis*	N/A	N/A	2.2	2.4	5.4	He/O_2_: 100:1, He through NaOCl
	Zhou et al. 2016 [52]	self-constr.	jet	0	Root canals	Yes	*E. faecalis*	4.9	6.1	7	N/A	N/A	He through H_2_O_2_
*Air*	Cao et al. 2011 [53]	self-constr.	jet	10	Cellulose	Yes	*E. faecalis*	N/A	N/A	N/A	2	N/A	
	Chang et al. 2016 [54]	self-constr.	DBD		Glass		*E. faecalis*	N/A	4	4.5	5.2	5.4	
	Theinkom et al. 2019 [55]	self-constr.	SMD	10	Agar	Yes	*E. faecalis*	7.9	N/A	8.6	9.1	N/A	
		self-constr.	SMD	10	Petri dish	Yes	*E. faecalis*	0	N/A	3.2	5.4	N/A	
		self-constr.	SMD	10	Petri dish	Yes	*E. faecalis*	1.8	N/A	2.6	5.7	N/A	
		self-constr.	SMD	10	Petri dish	Yes	*E. faecalis*	1.7	N/A	2.4	4.9	N/A	
	Ballout et al. 2018 [35]	Plasma Derm	DBD	2	Root canals	Yes	*E. faecalis*	0.1	N/A	N/A	N/A	N/A	
*Air + O_2_*	Zhou et al. 2016 [37]	self-constr.	jet	0	Root canals	Yes	*E. faecalis*	2.9	3.1	3.6	N/A	N/A	air through H_2_O_2_

**Table 4:** Effect of CAP on bacteria associated with periodontal disease; Abbreviations: conc.: concentration; DBD: dielectric barrier discharge; max red: maximal reduction; N/A: not available; nd: non-detectable; ns: non-significant; self-constr.: self-constructed; Ti: titanium.

Gas	Author	Device	Plasma mode	Distance (mm)	Surface	Biofilm	Species	Max Red t ≤ 60 s	Max Red t ≤ 120 s	Max Red t ≤ 300 s	Max Red t ≤ 600 s	Max Red t > 600 s
*Ar*	Abonti et al. 2016 [19]	self-constr.	jet	2	Agar	No	*A. actinomycetemcomitans*	ns (0.2)	N/A	N/A	N/A	N/A
		self-constr.	jet	20	Agar	No	*A. actinomycetemcomitans*	ns (0.3)	N/A	N/A	N/A	N/A
	Annunziata et al. 2016 [56]	Plasma R	DBD		Ti discs	Yes	*A. actinomycetemcomitans*	N/A	N/A	N/A	N/A	nd
	Carreiro et al. 2019 [57]	kINPen med	jet	7	Ti discs	Yes	*P. gingivalis*	1.2	N/A	1.2	N/A	N/A
*Air*	Liguori et al. 2017 [31]	self-constr.	DBD-Rod	3	Well Plate	No	*A. actinomycetemcomitans*	2.2	2.8	N/A	N/A	N/A
		self-constr.	DBD-Plate	3	Well Plate	No	*A. actinomycetemcomitans*	1.7	2.8	N/A	N/A	N/A
		self-constr.	DBD-Rod	3	Well Plate	Yes	*A. actinomycetemcomitans*	1	1.8	N/A	N/A	N/A
		self-constr.	DBD-Plate	3	Well Plate	Yes	*A. actinomycetemcomitans*	1.1	2	N/A	N/A	N/A
	Yang et al. 2018 [58]	self-constr.	jet	15	Agar		*P. gingivalis*	N/A	4	4.5	nd	N/A
*He*	Lee et al. 2019 [59]	self-constr.	jet	30	Ti discs	Yes	*P. gingivalis*	N/A	N/A	1.2	nd	N/A
*O_2_*	Abonti et al. 2016 [19]	self-constr.	jet	2	Agar	No	*A. actinomycetemcomitans*	1.9	N/A	N/A	N/A	N/A
		self-constr.	jet	20	Agar	No	*A. actinomycetemcomitans*	nd	N/A	N/A	N/A	N/A
*N_2_*	Abonti et al. 2016 [19]	self-constr.	jet	2	Agar	No	*A. actinomycetemcomitans*	ns (0.4)	N/A	N/A	N/A	N/A
		self-constr.	jet	20	Agar	No	*A. actinomycetemcomitans*	1.6	N/A	N/A	N/A	N/A
*O_2_ + N_2_*	Abonti et al. 2016 [19]	self-constr.	jet	2	Agar	No	*A. actinomycetemcomitans*	1.2	N/A	N/A	N/A	N/A
		self-constr.	jet	20	Agar	No	*A. actinomycetemcomitans*	1.2	N/A	N/A	N/A	N/A

**Table 5:** Effect of CAP on *C. albicans*; Abbreviations: DBD: dielectric barrier discharge; HDBD: hollow dielectric barrier discharge; max red: maximal reduction; PMMA: polymethylmethacrylate; N/A: not available; nd: non-detectable; ns: non-significant; self-constr.: self-constructed; Ti: titanium; VDBD: volume dielectric barrier discharge.

Gas	Author	Device	Plasma Mode	Distance (mm)	Surface	Biofilm	Species	Max Red t ≤ 60 s	Max Red t ≤ 120 s	Max Red t ≤ 300 s	Max Red t ≤ 600 s	Max Red t > 600 s	Additional
*Ar*	Handorf et al. 2018 [60]	kINPen 09	jet	18	Well-plate	Yes	*C. albicans*	1	1.5	2	N/A	N/A	
	Koban et al. 2010 [40]	kINPen 09	jet	7	Ti	Yes	*C. albicans*	0.4	0.5	0.5	0.3	N/A	
		self-constr.	HDBD	7	Ti	Yes	*C. albicans*	1.7	1.4	2	2.9	N/A	
		self-constr.	VDBD	15	Ti	Yes	*C. albicans*	2.3	2.2	3.5	5.2	N/A	
	Matthes et al. 2015 [61]	self-constr.	VDBD		PMMA	Yes	*C. albicans*	1.2	N/A	2.8	4.1	N/A	
		self-constr.	VDBD		PMMA	Yes	*C. albicans*	N/A	N/A	N/A	ns (0.1)	N/A	
		self-constr.	VDBD		PMMA	Yes	*C. albicans*	N/A	N/A	N/A	ns (−0.26)	N/A	
	Delben et al. 2016 [62]	kINPen	jet	10	Acrylic resin	Yes	*C. albicans*	1.7	N/A	N/A	N/A	N/A	
	Doria et al. 2019 [63]	self-constr.	jet		polyurethane	Yes	*C. albicans*	N/A	N/A	N/A	N/A	1.3	pulsed
		self-constr.	jet		polyurethane	Yes	*C. albicans*	N/A	N/A	N/A	N/A	1.5	continous
	Wanachantararak et al. 2019 [64]	self-constr.	jet	10	Agar		*C. albicans*	N/A	N/A	ns (0.1)	0.4	0.5	
*Ar + Air*	Doria et al. 2019 [63]	self-constr.	jet		polyurethane	Yes	*C. albicans*	N/A	N/A	N/A	N/A	1.1	Ar/Air: 1:9, continous
		self-constr.	jet		polyurethane	Yes	*C. albicans*	N/A	N/A	N/A	N/A	0.9	Ar/Air: 1:9, pulsed
		self-constr.	jet		polyurethane	Yes	*C. albicans*	N/A	N/A	N/A	N/A	1.9	Ar/Air: 6:4, continous
		self-constr.	jet		polyurethane	Yes	*C. albicans*	N/A	N/A	N/A	N/A	1.6	Ar/Air: 6:4, pulsed
*Ar + O_2_*	Kerlikowski et al. 2020 [65]	kINPen 08	jet	1–2	Root canals	Yes	*C. albicans*	N/A	N/A	N/A	2.1	2.7	Ar/O_2_: 100:1
	Koban et al. 2010 [40]	kINPen 09	jet	7	Ti	Yes	*C. albicans*	0.6	1	0.8	0.5	N/A	Ar/O_2_: 100:1
		self-constr.	HDBD		Ti	Yes	*C. albicans*	1.4	1.4	3	3.3	N/A	Ar/O_2_: 100:1
	Matthes et al. 2015 [61]	self-constr.	VDBD		PMMA	Yes	*C. albicans*	ns (0.6)	N/A	0.6	0.9	N/A	Ar/O_2_: 100:1
	Wang et al. 2016 [66]	self-constr.	jet	10	PMMA	Yes	*C. albicans*	1.9	2.6	3	5.9	N/A	Ar/O_2_: 98:2
*Air*	Maisch et al. 2012 [67]	self-constr.	SMD	6	Well-plate	No	*C. albicans*	5.5	nd	nd	nd	N/A	
		self-constr.	SMD	6	Well-plate	Yes	*C. albicans*	0.1	0.7	0.6	nd	N/A	
	Yoo et al. 2016 [68]	self-constr.	jet	3	PMMA	No	*C. albicans*	N/A	1.3	N/A	N/A	N/A	
*He*	Chiodi et al. 2017 [69]	self-constr.	jet	15	Well-plate	Yes	*C. albicans*	ns (0.3)	N/A	1.5	1.7	N/A	
	Song et al. 2012 [70]	self-constr.	brush		Glass	Yes	*C. albicans*	0.5	0.9	1	N/A	N/A	
	Doria et al. 2019 [63]	self-constr.	jet		polyurethane	Yes	*C. albicans*	N/A	N/A	N/A	N/A	1.1	continous
		self-constr.	jet		polyurethane	Yes	*C. albicans*	N/A	N/A	N/A	N/A	1.2	pulsed
*He + Air*	Doria et al. 2019 [63]	self-constr.	jet		polyurethane	Yes	*C. albicans*	N/A	N/A	N/A	N/A	2.6	He/Air: 6:4, continous
		self-constr.	jet		polyurethane	Yes	*C. albicans*	N/A	N/A	N/A	N/A	1.7	He/Air: 6:4, puseld
		self-constr.	jet		polyurethane	Yes	*C. albicans*	N/A	N/A	N/A	N/A	1	He/Air: 1:9, continous
		self-constr.	jet		polyurethane	Yes	*C. albicans*	N/A	N/A	N/A	N/A	1	He/Air: 1:9, pulsed
*He + O_2_*	He et al. 2020 [71]	self-constr.	jet	10	Well-plate	Yes	*C. albicans*	N/A	0.2	0.4	0.8	N/A	He/O_2_: 99.5:0.5
	Song et al. 2012 [70]	self-constr.	brush		Glass	Yes	*C. albicans*	0.4	0.8	1	N/A	N/A	He + O_2_ 1%
		self-constr.	brush		Glass	Yes	*C. albicans*	0.8	1.2	1.5	N/A	N/A	He + O_2_ 5%
		self-constr.	brush		Glass	Yes	*C. albicans*	0.5	1	1	N/A	N/A	He + O_2_ 7%
	Sun et al. 2012 [72]	self-constr.			Well-plate	Yes	*C. albicans*	nd	N/A	N/A	N/A	N/A	He/O_2_: 98:2
		self-constr.			Well-plate	Yes	*C. albicans*	nd	N/A	N/A	N/A	N/A	He/O_2_: 98:2
		self-constr.			Well-plate	Yes	*C. krusei*	nd	N/A	N/A	N/A	N/A	He/O_2_: 98:2
		self-constr.			Well-plate	Yes	*C. krusei*	nd	N/A	N/A	N/A	N/A	He/O_2_: 98:2
*He + O_2_ + N_2_*	Rupf et al. 2010 [33]	self-constr.	jet	1.5	Agar	Yes	*C. albicans*	5	N/A	N/A	N/A	N/A	He/O_2_/N_2_: 2:1.2:1.5
*N_2_*	Yoo et al. 2016 [68]	self-constr.	jet	3	PMMA	No	*C. albicans*	N/A	1	N/A	N/A	N/A	

## Data Availability

Data is contained within the article or supplementary material.

## Supplementary Materials

The following are available online at https://www.mdpi.com/2079-6382/10/2/211/s1, Table S1: Data extraction; Table S2: Quality assessment.
